# Cyberbullying roles and psychosocial dynamics: a latent profile analysis of loneliness, resilience, and self-regulation in adolescents

**DOI:** 10.1186/s12889-025-22745-w

**Published:** 2025-04-22

**Authors:** Sema Yazıcı-Kabadayı, Oğuz Mercan, Kemal Öztemel

**Affiliations:** 1 Recep Tayyip Erdogan University, Rize, Türkiye; 2 Ministry of Education, Ankara, Türkiye; 3 Gazi University, Ankara, Türkiye

**Keywords:** Cyberbullying roles, Loneliness, Resilience, Self-regulation, Adolescents, Latent profile analysis

## Abstract

Participation in cyberbullying roles is associated with distinct psychosocial profiles and may contribute to adverse mental health outcomes. Despite its importance, research has yet to fully explore the latent profiles associated with cyberbullying roles and their interplay with psychosocial factors among adolescents. This study explores the roles of cyberbullies, cyber victims, and cyber bystanders among adolescents, using latent profile analysis (LPA) to uncover the connections between these roles and psychosocial variables, including loneliness, resilience, and self-regulation. The study involved 394 adolescents, including 246 women and 148 men, with an average age of 15.8 years (SD = 1.04). The LPA revealed three profiles: low-risk, moderate-risk, and high-risk groups. The low-risk group (%64.1), characterized by minimal involvement in cyberbullying roles, had the highest levels of resilience and self-regulation and the lowest levels of loneliness. In contrast, those in the moderate-risk group (%27.8) showed moderate involvement in all cyberbullying roles, the highest levels of loneliness, and the lowest levels of resilience and self-regulation. The high-risk group (%8.2) demonstrated significant engagement across all cyberbullying roles and moderate levels of loneliness, resilience, and self-regulation. These findings underscore the protective role of resilience and self-regulation against cyberbullying, with loneliness as a potential risk factor, particularly for the moderate-risk group. In addition to highlighting the need for interventions that enhance resilience and self-regulation to prevent cyberbullying, the present study suggests that further research into the role of loneliness in cyberbullying profiles could provide valuable insights, inspiring future studies and furthering our understanding of this multifaceted issue.

## Introduction

The use of information and communication technologies has brought many advantages to humanity, but it has also brought some disadvantages. One of them is the existence and increase of cyberbullying in schools, which affects students, teachers, and parents [1]. Cyberbullying is a type of bullying and aggression [2], and cyberbullying, a particular form of bullying, has drawn considerable attention in recent years [3].

Cyberbullying is a systematic abuse of power that occurs using information and communication technologies [4] and can be defined as the intention to harass and harm another individual continuously through any electronic means, including social media. Cyberbullying includes behaviors such as assault and threats, humiliation, online verbal fights, cyber bystander, exclusion, sending material to harm someone, pretending to be someone else to publish, sharing embarrassing information or images of someone, creating fake profiles, and sharing someone's personal information [2]. Notably, fake profiles refer to fabricated identities that present false personal information, often to deceive and manipulate [5]. Empirical evidence suggests that adolescents who create fake profiles are at a heightened risk of engaging in cyberbullying-related activities [6]. Unlike other forms of online bullying, which may occur within specific timeframes or digital contexts, cyberbullying persists without temporal boundaries, amplifying its potential psychological impact on victims [7, 8]. Cyberbullying, which includes many different behaviors, involves different roles in terms of participation in these behaviors. Cyberbullying, which includes many different behaviors, involves different roles in terms of participation in these behaviors.

Cyberbullying involves people assuming various roles according to their involvement in the bullying process [9]. Therefore, in addition to focusing on behaviors, participant roles should also be considered [10]. Focusing on participant roles in cyberbullying will provide critical information for understanding the dynamics of cyberbullying and developing effective intervention strategies. Studies on cyberbullying have defined three dimensions of cyberbullying roles. These roles are defined as bully, victim, and bystander [11]. Bullies are those who exert power imbalances over others, persistently harass them, derive pleasure from their pain, and demonstrate diminished empathy. Children in the role of bullies have poor relationships and harbor hostility towards their environment. Victims are defined as children who are disadvantaged in terms of social relationships, passive, anxious, insecure, easily upset and have fewer friends. They have low self-esteem, do not report bullying for fear of reprisals, and have negative perceptions of themselves. The bystanders to bullying, in addition to the role of bullies and victims, are often overlooked, particularly children. When observing another child being bullied, bystanders can experience conflicting emotions, such as anger, sadness, fear, and indifference, even though the focus is on bullies and victims. They may also feel guilty for not being able to help their friends and fear that the same thing will happen to them. Bystanders show similar physiological reactions to victims. Bystanders show identical physiological responses to victims. Victims and bystanders may lose their sense of empathy over time and become desensitized to negative behaviors, leading to more problems [12]. Adolescents identified as bullies, victims, and bystanders show poorer psychosocial functioning, including suicidal ideation, internalizing/externalizing symptoms, isolation, aggression, emotional dysregulation, moral detachment, and social and emotional loneliness [13–15]. Evidence shows that adolescents can engage in different participant roles in the cyberbullying experience and become more prone to experiencing reduced social and forgiveness skills, aggressive behaviors, emotional problems, and psychological disorders related to these roles [16]. In previous studies, cyberbullying, a significant public health issue affecting young people, requires monitoring by parents, school authorities, and mental health professionals for potential effects and intervention strategies [17]. Recent studies have also highlighted cyberbullying as a significant public health issue that poses a threat to individuals' development, particularly during childhood and adolescence [18, 19].

Cyberbullying is especially prevalent among individuals in developmental stages, such as adolescence, and can lead to psychological effects that may persist into later stages of life. For this reason, cyberbullying is quite common due to the nature of online interaction, and its effects are long-term [20]. Their international study on adolescence [21] found the prevalence of cyberbullying to be 30.5%. In one of the latest studies, this rate was 42.8% [22]. The prevalence of cyberbullying among adolescents in Türkiye was found to be 65.3% [23]. Another study conducted in Türkiye found that 35.7% of students exhibited bullying behaviors, 23.8% exhibited bully-victim behaviors, and 5.9% of students were victims [24]. Based on the results of studies conducted, it is possible to say that cyberbullying is a critical problem area and has the potential to affect a large segment of society. When asked directly whether they were actively or passively involved in bullying via the internet or mobile phone, most participants (63.8%) believed cyberbullying was a “big problem.” Additionally, 61.9% reported being a victim, 52.5% a perpetrator, and 76.3% a bystander at least once within three months. The most common types of bullying are insulting or threatening, deceiving, spreading gossip via the internet or mobile phone, and entering someone's computer and changing their password [25]. In addition to the data regarding the prevalence and severity of cyberbullying, some research findings indicate that victims of cyberbullying experience emotional challenges such as depression, anxiety, and low self-esteem [26].

Another study showed the most common perpetrators of cyberbullying and found a positive relationship between cyber-victim and cyberbullying perpetration [27]. Adolescents who are cyber-victimized are more likely to engage in cyberbullying behaviors than those who are not cyber-victimized [28]. Most teenagers choosing to be bystanders suggests that emphasizing bystander involvement, like addressing traditional bullying issues, could significantly enhance bullying prevention efforts [29]. An increasing number of adolescents report being cyberbullied in different bullying roles, making cyberbullying an increasingly important problem. Anonymity and lack of control over the digital environment exacerbate this social threat, often leading to serious negative consequences [30]. Understanding cyberbullying's psychological impact requires studies that consider all bullying roles together. In addition, investigating the psychosocial factors associated with bullying roles is extremely important in forming the basis for developing and implementing preventive and intervention programs in schools.

### Loneliness

Loneliness, which refers to the inconsistency between the relationships one wants to have and the relationships one has [31], is defined as subjectively perceived social isolation [32] and is considered an indicator of the social relationship difficulties one experiences [33]. Adolescents who experience social problems may be more at risk of cyberbullying [34].

Loneliness is one of the variables thought to be related to cyberbullying because cyberbullying most commonly occurs in the context of relationships [35]. Cyberbullying begins to occur more frequently in childhood, especially with the social relationships that develop during adolescence, and continues to manifest itself even at later stages of adolescence [34]. Adolescence is a critical period when the importance of relationships begins to shift from family to peers, and it can be stated that a higher level of peer support against loneliness will reduce cyberbullying victimization [36]. Studies are showing that cyberbullying is associated with loneliness [37–39]. A study conducted on adolescents in Türkiye showed a significant relationship between cyber victimization and loneliness among adolescents [38]. Loneliness has been found to predict both cyberbullying victimization and cyberbullying perpetuation in adolescents [40]. The findings reveal that we should consider peer groups more likely to be cyber bystanders in the broader social context. We should also focus on the issue of loneliness [14]. Thus, loneliness is a risk factor for cyberbullying roles. The potential of loneliness to negatively affect individuals' coping skills in terms of cyberbullying roles shows that it should be considered an essential variable in terms of cyberbullying.

### Resilience

Resilience is a dynamic phenomenon that enables people to be relatively more resilient in overcoming stress or adversity in the face of environmental risk factors and to achieve relatively good outcomes compared to people who are not resilient despite significant stressors [41, 42]. Resilience also refers to the ability to adapt in the face of stress and adversity [43]. Cyberbullying is defined as a phenomenon that can harm the mental health and academic success of young people, and resilience prevents individuals from being victimized or less affected by cyberbullying and prevents the deterioration of their psychological well-being [44].

Despite several studies examining adolescents' experiences of bullying, few studies have addressed how young people manage these risk situations and develop resilience [45, 46]. In contrast, we know that resilience is a factor that protects young people from specific harms when they are bullied or cyberbullied [47]. Resilience is a vital intervention area for the prevention of cyberbullying and for adolescents to manage their bullying behaviors [48]. Existing studies focus on the roles of bullies and victims of cyberbullying. However, there is limited research on psychological resilience, which can be considered a practical personal resource in coping with cyberbullying [49] and overlook the bystanders. This situation creates the need to address resilience in terms of the different roles of cyberbullying.

### Self-regulation

Self-regulation is the capacity to gather information, consider options and consequences, make decisions, consider emotional factors associated with the decision-making process, set adaptive goals, and make adaptive choices to achieve a specific goal [50]. Considering that it includes the capacity to control one's behaviors and to perform activities that serve one's wants and needs [51], self-regulation is an important concept related to cyberbullying. Self-regulation is also crucial in cyberbullying roles. Studies are showing that cyberbullying is associated with self-regulation [52, 53] in adolescents. Self-regulation prevents aggressive defensive intervention by cyber bystanders in cyberbullying incidents [53]. Adolescents with poor self-regulation levels are more likely to show cyberbullying behaviors because they cannot control their impulses and cannot think about the future consequences of their actions [54]. Therewithal, those with low self-regulation are more likely to engage in cyberbullying behavior than those with high self-regulation [55]. Self-regulation can effectively ensure that adolescents seek the right support resources and use coping strategies such as reporting bullying [56]. Young people need to develop competencies that can effectively avoid or reduce bullying. Youth with poor self-regulation skills may be less likely to achieve goals and achieve social goals, which may lead to problems with bullying [57]. In this respect, self-regulation is thought to be an important variable associated with cyberbullying roles.

In line with the research findings on the three concepts of loneliness, resilience, and self-regulation, cyberbullying and cyberbullying profiles are also theoretically interconnected and linked to these three concepts. At this point, the social-emotional learning approach is an approach that includes concepts such as self-control, self-monitoring, and self-awareness, which play a protective role against possible risks in individuals' lives [58]. Similarly, resilience, which finds its place within the Positive Youth Development (PYD) structure, encourages the development of positive behaviors and plays a protective role against risky behaviors [47]. Finally, within the framework of Socio-Ecological Theory [59], cyberbullying victimization is understood as a consequence of complex social interactions, wherein factors such as loneliness and social connectedness play a critical role in shaping individuals' vulnerability to or resilience against such experiences.

### Current study

From the past to the present, cyberbullying has become an increasing threat that negatively affects the psychosocial development of individuals, especially those in adolescence, and prevents them from living their lives during their developmental period [60]. Based on this threat, adolescents who are exposed to cyberbullying face vital problems such as depression, anxiety, and low self-confidence [61] and also research shows that cyberbullying has become a widespread problem among adolescents [62], reaching dimensions that threaten mental health [63] and well-being [64] and is associated with depressive affect [65], anxiety [66], stress [67], loneliness [40], suicidal thoughts [68] and somatic symptoms [69, 70]. The fact that cyberbullying is increasing and continues to affect the mental health of adolescents negatively has made it necessary to develop intervention strategies further [2]. In this context, the most innovative and successful approach to cyberbullying is identifying bullies, victims, and bystanders before this situation occurs [16]. Within this framework, identifying the factors that contribute to involvement in cyberbullying behaviors, along with ensuring the early detection of cyberbullying and developing effective intervention strategies, is crucial for promoting both individual mental health and societal well-being [8, 71]. The studies provide valuable insights into the relationships between various variables, yet they often neglect to explore the rich tapestry of individual similarities and differences that can significantly influence outcomes. In summary, there is a need for comprehensive approaches that address all the roles associated with cyberbullying, including not only bullies and victims but also bystanders. By addressing these roles, we want to provide resources for intervention and treatment programs designed for individuals with different profiles and to reveal risk factors and preventive factors for adverse consequences of cyberbullying in terms of mental health.

The profile of the characteristics associated with adolescents' cyberbullying roles in terms of personal and social factors is essential. Information gathered through variable-centered approaches is often inadequate for understanding individuals' characteristics. In contrast, person-centered approaches analyze behavior within a holistic framework. This method looks at an individual based on patterns of personal factors related to the specific issue being studied [72]. This perspective elucidates how variables are integrated and processed collaboratively within each individual [73]. Based on construct-based identification, LPA treats constructs as profiles over unobservable categorical variables [74]. LPA categorizes individuals into distinct groups based on similar response patterns in social sciences. This technique uncovers unique behavioral subgroups within a sample, which may be overlooked in variable-centered analyses. By identifying these subgroups, LPA enables researchers to explore significant individual differences and common behavioral patterns more thoroughly in social science research [75]. In social sciences, it is essential to identify both typical and atypical patterns. In a variable-focused approach, it is more difficult to obtain a realistic view of complex dynamic systems of individuals [72]. This aspect of person-centered methods is crucial for uncovering insights that apply to a particular group, a significant objective in the social sciences [76]. Identifying significant subgroups in this way has prevention and treatment implications [77]. Approaches that acknowledge the existence of different profiles of cyberbullying roles may provide unique insights into the effects of profiles of cyberbullying roles on other variables. The study used LPA, a method used to identify types or groups of individuals with different structural profiles. LPA was used in this study as it helps define profile indicators as sub-populations that differ qualitatively and quantitatively [78]. Accordingly, this study aims to create psychosocial profiles of cyberbullying roles among adolescents, who are a risky group in terms of cyberbullying roles. Identifying cyberbullying profiles using Latent Profile Analysis (LPA) offers advantages over traditional variable-centered analysis methods. First, LPA enables classification based on common characteristics, which helps to differentiate behavioral patterns within diverse samples. This approach allows for the identification of not only individual differences in cyberbullying roles but also the psychosocial factors that underline these roles and their interactions. While variable-centered analyses often overlook individual differences within groups, person-centered approaches provide detailed insights into each group's dynamics and risk factors. As a result, intervention programs and prevention strategies can be tailored to both general trends and the specific needs of identified subgroups. This contributes to the development of more targeted and effective interventions, particularly for individuals at risk in the fight against cyberbullying.

Previous research has provided correlational and descriptive results on cyberbullying roles, but profile studies in which all bullying roles are addressed with various psychosocial variables are limited. There are few studies of latent profile analyses for cyberbullying during adolescence. Some studies addressed bullying victimization in adolescents using LPA [79]. The study conducted on South Korean adolescents found three cyberbullying profiles: high-risk, temporary, and low-risk [80]. Another study of Spanish adolescents [81] with latent class analysis (LCA) revealed the existence of three profiles regarding cyberbullying: The first profile was defined as "not" because it had shallow scores on the cyberbullying subscales. Researchers described the second profile as "Bully-victims"; they defined the third and final profile as "Rarely victims and bullies". However, in cyberbullying-based research in adolescence, it is emphasized that there are four profiles regarding cyberbullying (cyberbullies, cyber victims, victim cyberbullies, and other profiles other than these profiles) [82]. It is crucial to address the specific characteristics of cyberbullying during adolescence from this perspective. Hypothesis 1 suggests that potential subgroups of cyberbullying roles can be observed.

Self-regulation, loneliness, and resilience play an important role in adolescents' bullying roles. Self-regulation emerges as a protective factor against bullying in early adolescence [57], and similarly, resilience plays a pivotal role in adolescent development, particularly in the context of the digital age [83]. In addition to its protective and significant roles, emotional factors such as loneliness are also emphasized in cyberbullying profiles among adolescents [84]. Despite the aforementioned research findings and the considerable importance ascribed to them, the previous research has been limited because it has not yet examined the relationship between risk and protective factors in terms of profiles of adolescents' cyberbullying roles. The role points to specific personal resources that need empowerment in addressing adolescent bullying roles. The role point indicates the personal resources that require empowerment in addressing adolescents' bullying roles. The current study may contain differences in levels of bullying roles and psychosocial factors in the profiles. Using LPA, we aimed to examine the relationships between cyberbullies, cyber victims, cyber bystanders, self-regulation, resilience, and loneliness. Based on this purpose, Hypothesis 2 suggests that subgroups of cyberbullying roles (cyberbully, cyber victim, cyber bystander) are associated with loneliness, self-regulation, and resilience. In this sense, we focused on the profiles mentioned based on cyberbullying and based on measurements taken through LPA. For this purpose, we started the data collection process by contacting the schools where volunteer participants studied in the first semester of 2024 (more detailed information in the 2.4. Procedure and Data Analysis section). After this stage, the researchers classified the participants using a participant-centered approach and aimed to compare the profiles regarding bullying, cyber victimization, cyber bystander, self-regulation, resilience, and loneliness. To examine the data, we used LPA to explore the profiles, focusing on the characteristics of different model subgroups. The current study proposed the following hypotheses: (1) potential subgroups of cyberbullying roles can be observed; (2) subgroups of cyberbullying roles (cyberbully, cyber victim, cyber bystander) are associated with loneliness, self-regulation, and resilience.

## Method

### Ethical considerations

Approval was obtained from the Ethics Committee of Recep Tayyip Erdoğan University. Informed consent was obtained from the participants before data collection, and data confidentiality was ensured by not reporting the participants' identities and reporting the findings only collectively. Participants were informed that participation in the study was voluntary and that they could withdraw from the study at any time.

### Participants

Four hundred eighteen adolescents were surveyed using convenience sampling for this study. Participants were 394 adolescents (*n* = 246, 62,4% women and *n* = 148, 37,6% men) selected by convenience sampling method from Türkiye. All participants were public school students. The ages of the participants ranged from 13 to 19 years (M_age_ = 15,8, SD = 1,04). Participants were 9th grade (*n* = 92, 23,4%), 10th grade (*n* = 147, 37,3%), 11th grade (*n* = 140, 35,5%) and 12th grade (*n* = 15, 8%). The analysis of the sleep duration of the participants revealed that the majority (72.3%) slept for 6–8 h, while a minority (10.9%) slept less than 6 h, and a significant portion (16.8%) slept more than 8 h. Additionally, most participants (84%) reported using the internet for more than 2 h, with only 16% indicating internet usage between 0–2 h.

### Measures

#### Cyber Bullying Scale

The researchers measured the cyberbullying roles of adolescents using the Cyber Bullying Scale. This scale consists of three dimensions, with 14 items in each dimension and 42 items in total. Adolescents answer the items from 0 to 3 (from never true to always). Cyber Bullying Scale adapted for adolescents in Türkiye [85]. For the Turkish adolescents, the three-dimensional structure showed a good fit with the data. The fit indices found in the adaptation study from the confirmatory factor analysis were χ2/sd = 3.51, RMSEA = 0.08, RMR = 0.02, SRMR = 0.06, GFI = 0.91, CFI = 0.97. The analysis conducted for reliability studies found the internal consistency coefficient to be 0.88 for the victim, 0.90 for the bully, and 0.93 for the bystanders [85]. The estimated Cronbach's alphas of the total scale score and three subscales ranged from 0.80 to 0.90; McDonald's ω of the total scale score and three subscales ranged from 0.80 to 0.90 in the current sample. The CFA results of the cyberbullying scale in the current study showed that the fit indices were satisfactory in the sub-dimensions of cyber victim (CFI = 0.88, GFI = 0.92, RMSEA = 0.079, SRMR = 0.062), cyber bully (CFI = 0.90, GFI = 0.93, RMSEA = 0.078, SRMR = 0.062) and cyber bystander (CFI = 0.94, GFI = 0.98, RMSEA = 0.064, SRMR = 0.038).

#### Short form of the UCLA loneliness scale

The UCLA Loneliness Scale measured the loneliness of adolescents. This scale consists of one dimension, with seven items total. Adolescents answer the items from 1 to 4 (from nothing true to always). UCLA Loneliness Scale adapted for adolescents in Türkiye [86]. For Turkish adolescents, the one-dimensional structure showed a good fit with the data. The analyses conducted for reliability studies found the internal consistency coefficient to be 0.74 [86]. The estimated Cronbach's alphas of the total scale score are 0.74; McDonald's ω 0.75 in the current sample. The CFA results in the present study showed that the scores of the fit indices were satisfactory (CFI = 0.98, GFI = 0.99, RMSEA = 0.053, SRMR = 0.030).

#### Self-Regulation Scale for Adolescents

The self-regulation of adolescents was measured using the Self-Regulation Scale for Adolescents. This scale consists of one dimension, with 11 items in total. Adolescents answer the items from 1 to 5 (from strongly disagree to true to agree strongly). Self-Regulation Scale developed for adolescents in Türkiye [87]. The confirmatory factor analysis in the developing study found the fit indices: χ2 / Sd = 4.55; CFI = 0.93; TLI = 0.91; SRM*R* = 0.041; RMSEA = 0.089. The analyses conducted for reliability studies revealed an internal consistency coefficient of 0.90 [87]. The estimated Cronbach's alphas of the total scale score are 0.87; McDonald's ω 0.87 in the current sample. The CFA results in the present study showed that the scores of the fit indices were satisfactory (CFI = 0.94, GFI = 0.99, RMSEA = 0.079, SRMR = 0.045).

#### Child and Youth Resilience Measure (CYRM-12)

The researchers measured the adolescents' resilience using the Child and Youth Resilience Measure (CYRM-12). This scale consists of one dimension, with 12 items in total. Adolescents answer the items in the range of 1 to 5 (from It does not define me at all valid to It ultimately defines me). CYRM-12 was developed for adolescents in Türkiye [88]. The fit indices found in the adopting study's confirmatory factor analysis were χ2 / Sd = 2.03; GFI = 0.94; NFI = 0.94; CFI = 0.97; IFI = 0.97. As a result of the analyses conducted for reliability studies, the internal consistency coefficient was found to be 0.91 [88]. The estimated Cronbach's alphas of the total scale score are.82; McDonald's ω 0.82 in the current sample. The CFA results in the present study showed that the scores of the fit indices were satisfactory (CFI = 0.92, GFI = 0.99, RMSEA = 0.065, SRMR = 0.045).

### Procedure and data analysis

The Recep Tayyip Erdoğan University Ethics Committee approved the study (No: 2024/043, February 14, 2024). The data collection process was conducted face-to-face by obtaining research permission from the schools affiliated with the Ministry of National Education. All participants were informed about the purpose of the study. The participants who volunteered and provided informed consent completed the scale set. The school management requires all questionnaires to be completed within a lesson hour, equivalent to forty minutes. Participation was voluntary, and no personal information was collected.

In a person-centered approach, removing any missing data is advisable to ensure the integrity of the analysis [72]. Before initiating the analysis, the data set underwent a thorough examination to check for completeness, and it was confirmed that no missing data points were present. Outliers were detected using the skewness-kurtosis test [89]. We excluded the 24 identified outliers from the dataset. The initial number of participants (*N* = 418) was reduced to *N* = 394 because 24 participants were outliers. It is recommended in the literature that the required sample size for LPA should be in the range of 300–500 [75]. We tested the validity of our scales after reaching a sufficient sample size of 394 participants in the study, which was in an adequate range. At this stage, we performed analyses using SPSS and JASP.

Our main objective was to generate a typology of cyberbullying roles using the LPA method and to validate the types using analyses of variance (ANOVAs) and post hoc comparisons. LPA was executed in Mplus to explore latent profiles based on a combination of cyberbullying roles with loneliness, self-regulation, and resilience. We used the Bayesian Information Criterion (BIC), Sample Adjusted Bayesian Information Criteria (SABIC), Akaike Information Criterion (AIC), Lo–Mendell–Rubin adjusted likelihood ratio test (LMR), Bootstrapped likelihood ratio test (BLRT) and entropy test fit indices to select the optimal number of profiles [90]. The researchers conducted analyses of variance (ANOVA) to test the presence of a significant difference between the three profiles.

## Results

### Preliminary analyses and correlations

Table 1 shows the correlation coefficients among variables, descriptive statistics, and Cronbach’s alpha coefficients. All correlation coefficients were significant at the *p* < 0.01 and *p* < 0.05. A measurement model was run in six variables.

**Table 1:** Preliminary analysis and correlations

Variable	M	SD	Skewness	Kurtosis	1	2	3	4	5	6
1 Cyber victim	16.21	3.24	2.29	6.41	–					
2Cyber bystanders	18.87	5.99	1.71	3.22	.52^**^	–				
3 Cyberbullying	15.07	2.38	2.97	9.52	.50^**^	.34–	–			
4 Loneliness	13.29	4.55	.49	-.65	.11^*^	.06	.11^*^	–		
5 Resilience	45.22	8.17	-.37	-.33	-.28^**^	-.20^**^	-.23^**^	-45^**^	–	
6 Self-regulation	42.71	7.53	-.47	.20	-.19^**^	-.06	-.12^*^	-.25^**^	.56^**^	–

*N* = 394, ^**^p < . 01, ^*^p < .05

Table 1 shows the descriptive statistics and correlation results. Kline [89] indicates that in social sciences, kurtosis values for a normal distribution typically range from + 3 to -3, while skewness values range from + 10 to -10. Based on these guidelines, the skewness and kurtosis values of the variables were analyzed, confirming that the data exhibited a normal distribution. This finding validates the research since the values fell within the ranges established by Kline [89]. Cyber victims are positively related to cyber bystanders (*r* = 0. 52, *p* < 0.01), cyberbullying (*r* = 0. 50, *p* < 0.01), and loneliness (*r* = 0.11, *p* < 0.05). Cyber victims are negatively related to resilience (*r* = -0.28, *p* < 0.01) and self-regulation (*r* = -0.19, *p* < 0.01). Cyber bystanders negatively related to resilience (*r* = -0.20, *p* < 0.01). Cyberbullying is negatively related to resilience (*r* = -0.023, *p* < 0.01) and self-regulation (*r* = -0.12, *p* < 0.05). Also, cyberbullying is positively related to loneliness (*r* = 0.11, *p* < 0.05). Loneliness is negatively related to resilience (*r* = -0.45, *p* < 0.01) and self-regulation (*r* = -0.45, -0. 25, *p* < 0.01). Self-regulation is positively related to resilience (*r* = 0.56, *p* < 0.01). All variables were found to be related to being a cyber victim and cyber bully. In contrast, only resilience was found to be related to cyber bystanders among the roles of cyberbullying.

### Common method bias

Common method bias can occur when dependent and independent variables are measured using the same response method in social sciences, potentially undermining the validity of studies [91]. Therefore, it is essential to control for common method bias. Harman's single-factor test is designed to address this issue in survey research [92]. This study analyzed the research data using Harman's single-factor test. The findings indicated that all variables accounted for 14.18% of the variance in the first factor. Since this percentage is below the 50% threshold, the results were considered free of standard method bias [93].

### LPA

**Fig. 1:**
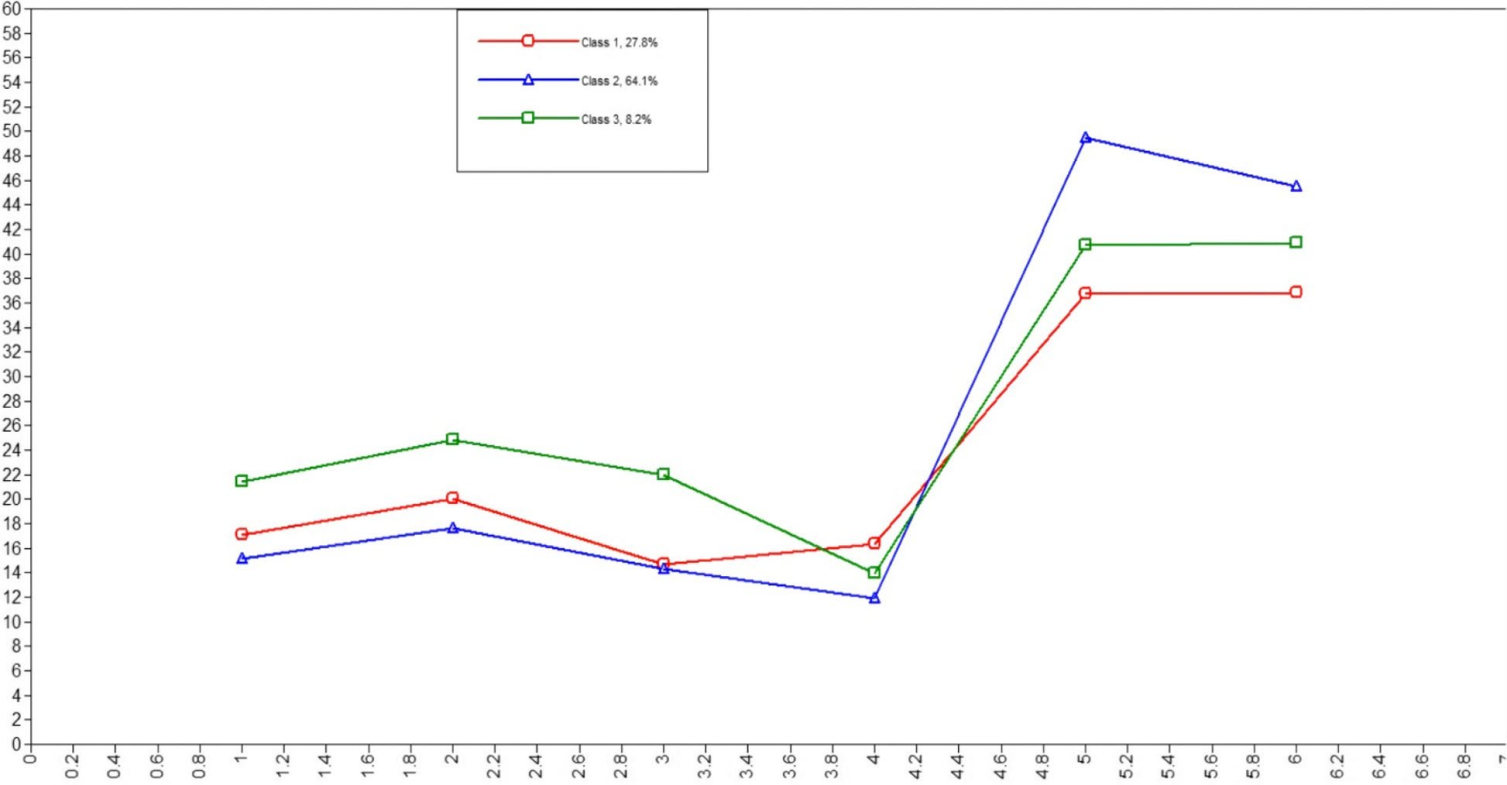
Three-profiles model. *Note.* 1 = Cyber victim, 2 = cyber-bystander, 3 = cyberbullying, 4 = loneliness, 5 = resilience, 6 = self-regulation

An LPA was conducted with Mplus to identify the potential profiles of adolescents’ cyberbullying roles. LPA models ranged from two to five profiles [90]. The indices for the LPA and profile structure are shown in Table 2. Lower BIC and AIC indicate better model fit, and LMR and BLRT require a significant* p*-value. Entropy, on the other hand, is related to the precision in classifying respondents, and values above 0.80 are considered acceptable. The AIC, BIC, and SABIC values decreased from one-profile to five-profile models. The LMR (p) value showed that a five-profile solution was more appropriate than a two- and three-profile solution, but a four-profile solution was not appropriate than a three-profile solution. The BLRT (p) value was significant for all models. The entropy value was highest for the four-profile model and decreased for the two-profile, five-profile and three-profile models. The lowest group percentage and fit indices indicated that the three-profile model gave the most appropriate answer for grouping adolescents. Therefore, the three-profile model was the optimal solution. Figure 1 shows the profile characteristics of cyberbullying roles.

**Table 2:** Latent profiles

Model	Parameter Numbers	AIC	BIC	SABIC	Entropy	LMR	BRLT	Lower Group Percentage
1	12	14191.491	14239.20	14201.131	-	-	-	-
2	19	13738.659	13,814.210	13753.923	.982	0.0154	0.0000	%9.1
***3***	***26***	***13559.351***	***13662.736***	***13580.238***	***.842***	***0.0298***	***0.0000***	***%8.2***
4	33	13345.459	13476.679	13371.970	.988	0.0576	0.0000	%5.3
5	40	13218.700	13377.754	13250.835	.884	0.0011	0.0000	%1.5

### Comparing cyberbullying profiles

One-way ANOVA was employed to examine whether there were differences among the identified profiles in terms of their cyber victim, cyberbullying, cyber bystanders, loneliness, resilience, and self-regulation (Table 3). Table 3 shows the results of the ANOVAs with post-hoc comparisons.

**Table 3:** Means and standard deviations for the three-profile model (*N* = 394)

	Profile 1 Moderate-risk		Profile 2 Low-risk		Profile 3 High-risk		F	*p*
Variables	M	SD	M	SD	M	SD		
Cyber victim	17.25	3.33	15.13	1.72	21.21	3.24	86.69	< .001
Cyber-bystanders	20.07	6.58	17.63	5.00	24.84	6.91	26.68	< .001
Cyberbullying	14.75	1.24	14.35	.85	21.97	2.72	566.87	< .001
Loneliness	16.47	4.36	11.88	3.80	14.06	5.37	47.67	< .001
Resilience	35.97	4.87	49.61	5.19	40.81	8.51	246.24	< .001
Self-regulation	36.71	7.13	45.55	5.87	40.88	8.68	69.241	< .001

*M* Mean, *SD* Standard deviation

Profile 1 includes adolescents who reported moderate levels of cyberbullying (M = 14.75, SD = 3.33), cyber-bystanders (M = 20.07, SD = 6.58), and cyber victim (M = 17.25, SD = 3.33) roles, high levels of loneliness (M = 16.47, SD = 4.36), and low levels of self-regulation (M = 36.71, SD = 7.13) and resilience (M = 35.97, SD = 4.87). The term "moderate-risk group" might be appropriate for this profile. Profile 2 (64.1%) includes adolescents who see themselves in the below-average roles of cyberbully (M = 14.35, SD = 0.85), cyber victim (M = 15.13, SD = 1.72), and cyber-bystanders (M = 17.63, SD = 5.00), have an average level of loneliness (M = 14.06, SD = 3.80), and have the highest level of self-regulation (M = 40.88, SD = 8.68) and resilience (M = 40.81, SD = 8.51). This profile may be characterized as the "low-risk group." Adolescents who were members of Profile 3 (8.2%) had the highest levels of cyberbullying (M = 21.97, SD = 2.72), cyber victim (M = 21.21, SD = 3.24) and cyber-bystanders (M = 24.84, SD = 6.91) and had average loneliness (M = 14.06, SD = 5.37), self-regulation (M = 40.88, SD = 8.68) and resilience (M = 40.81, SD = 8.51) scores. The term "high-risk group" can be used to describe this profile.

Post-hoc (Scheffe) results revealed that Profile 1 (moderate-risk group) had higher scores on the cyber bystanders, cyber victims, cyberbullying, and loneliness than Profile 2 (low-risk group) (*p* < 0.001). However, Profile 1 (moderate-risk group) had lower scores on resilience and self-regulation than Profile 2 and Profile 3 (high-risk group) (*p* < 0.001). Profile 2 had higher scores on the resilience and self-regulation scale than Profile 3 and Profile 1 (*p* < 0.001). Similarly, as expected, Profile 3 had higher scores on the cyber victim, cyber bystanders, and cyberbullying than Profile 1 and Profile 2 (*p* < 0.001).

**Fig. 2:**
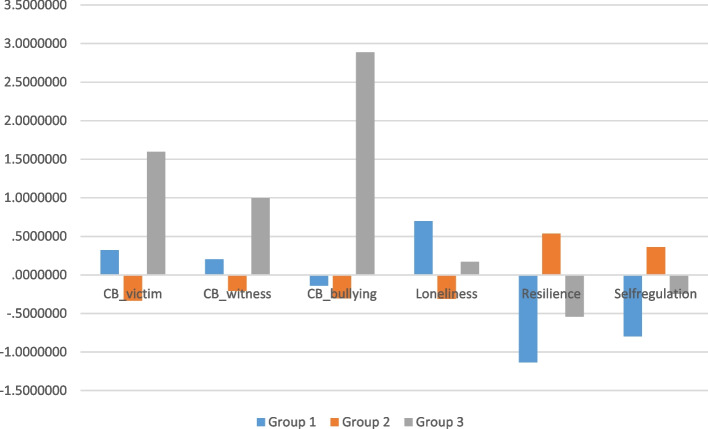
Post-hoc results of model variables

To analyze the outcomes of the three profiles, we compared the differences among adolescents ' cyberbullying profiles and the related variables of each profile (See Fig. 2). The low-risk group exhibited higher levels of loneliness but showed the lowest mean scores for cyber victimization, cyberbullying, and cyber bystanders. This group also had the highest scores for resilience and self-regulation. Conversely, the high-risk group had the highest scores for being cyber victims, cyber bullies, and cyber bystanders compared to all other groups. However, their scores for loneliness, self-regulation, and resilience were comparable to the averages of the other groups. In the moderate-risk group, scores for being cyberbullies, cyber victims, and cyber bystanders were around the average of the other groups, while their resilience and self-regulation scores were the lowest. The individuals in the low-risk group exhibited the highest mean scores for psychological resilience and self-regulation. Surprisingly, these scores were not followed by those in the moderate-risk group. In summary, the self-regulation skills and resilience levels were highest in the low-risk group of cyberbullying profiles. The moderate-risk group exhibited the lowest scores in resilience and self-regulation and the highest levels of loneliness.

## Discussion

The first aim of this study was to identify the different cyberbullying profiles by the potential subgroups of cyberbullying roles (cyberbully, cyber victim, and bystander), loneliness, resilience, and self-regulation in adolescents. To investigate these two questions, we used LPA, a person-centered approach.

Based on Hypothesis 1 in this study, three profiles related to cyberbullying were identified through the analysis of latent profiles: the first profile, with reported below-average roles of cyberbullying, cyber bystanders, and cyber victims, average levels of loneliness, and the highest level of self-regulation and resilience, was identified as a “low-risk group.” The second profile, with reported moderate levels of cyberbullying, cyber bystanders, and cyber victim roles, high levels of loneliness, and low levels of self-regulation, was identified as a “moderate-risk group.” The last profile, with reported highest levels of cyberbullying, cyber bystanders, and cyber victim roles, average levels of loneliness, self-regulation, and resilience, was identified as a “high-risk group.” These profiles obtained in the current study are consistent with the research results that reveal three profiles regarding cyberbullying using the latent analysis techniques [80–82]. In a study conducted using this technique [82] identified three groups based on levels of cyberbullying and victimization: not involved, victimized cyberbullies (moderate levels), and cyberbullies (high levels). Similarly, identified a high cyberbullying group with high aggression and victimization, a low group with moderate levels, and a non-cyberbullying profile [81]. In another study also found three profiles: high-risk, temporary, and low-risk [80]. This study’s findings align with these previous works, identifying high-risk, moderate-risk, and low-risk profiles based on varying levels of involvement in cyberbullying roles. Additionally, the high-risk, moderate-risk, and low-risk profiles identified in this research correspond to the profiles observed in some studies [80, 81] showed distinct categories based on the intensity of cyberbullying roles.

Findings obtained within the scope of this study are that all three roles of cyberbullying are present in all profiles. In the profiles that emerged in our study, it was seen that someone in the victim role also received similar scores in terms of bullies or bystander roles. This finding can be interpreted as being a cyberbully, cyber victim, or cyber bystander, which may also be related to other roles. Research findings indicate that those who take on one role of bullying are likely to take on other roles as well [94, 95] In these roles of bullies and victims are not mutually exclusive [96]. This is especially true in adolescence, where these roles can interact and create various roles, such as the bully-victim role. Similarly, there are also studies showing that cyber-victim roles predict the level of cyberbullying [97] and the relationship between cyberbullying and cyber-victimization [98–100]. Another study highlights that [101] in both bullying and cyberbullying, bystanders play a crucial role alongside aggressors and victims, and individuals often occupy multiple roles—such as bully, victim, or bystander—indicating an interrelated and cascading dynamic between these roles [25]. This scenario demonstrates that the roles of victim, bystander, or bully in cyberbullying are not easily distinguishable. As a result, it can be argued that certain aspects are susceptible to comparable risks in terms of cyberbullying roles and should be carefully considered in this context. In this sense, LPA helped define profile indicators as a subpopulation in the current study [78] and showed that being a cyberbully, victim, or witness to bullying during adolescence may increase the likelihood of taking on different roles later in life.

Hypothesis 2 in this study was that subgroups of cyberbullying roles (cyberbully, cyber victim, cyber bystander) would be associated with loneliness, self-regulation, and resilience. Our study found moderate levels of loneliness in the high-risk group, high levels in the moderate-risk group, and low levels in the low-risk group. Studies have shown that cyberbullying is associated with loneliness and that this can have long-term effects [4, 34, 38, 40, 84, 102, 103]. Loneliness helped differentiate between bullying profiles using LPA, and loneliness could aid in the early detection of bullying [104]. Also, high loneliness scores have been observed among adolescent cyber victims [84], while low social relationships and loneliness are risk factors for cyber bystanders [105]. Loneliness appears to be a significant factor across different cyberbullying roles, with higher levels of loneliness associated with an increased risk of involvement in cyberbullying, either as a victim or bystander. These findings suggest that loneliness is present across all cyberbullying profiles and remains an important variable within these profiles. Consistent with prior research, our findings suggest that loneliness may not only differentiate between cyberbullying profiles but also serve as an early indicator of vulnerability to these roles, highlighting its importance for targeted interventions. Our findings are consistent with the research results, but we also found that loneliness had similar mean scores across all groups. This result may be due to reasons such as the importance of loneliness in adolescence in developing social relationships and the rapid increase in the rate of loneliness among adolescents worldwide in recent years [106]. This suggests that the role of loneliness in cyberbullying profiles requires further examination. It is essential to evaluate profiles related to cyberbullying within a broader social context. The issue of loneliness, when examined within the scope of social contexts, is an essential variable in explaining cyberbullying among individuals during adolescence [14]. Although our study is cross-sectional and does not allow us to establish the temporal nature of the relationship between loneliness and cyberbullying profiles, the differences in loneliness scores across profiles suggest that these variables are closely linked and may mutually affect each other.

Another point to emphasize in this study is that we compared the differences between the cyberbullying profiles of adolescents and the relevant variables of each profile to analyze the results of the three profiles. Our finding was that the low-risk group showed higher levels of loneliness but the lowest mean scores for cyber victimization, cyberbullying, and cyber bystanders. We also found that this group had the highest scores for resilience and self-regulation. Previous research has highlighted resilience as a protective factor for cyberbullying [45–47]. Findings reveal that cyberbullying and victimization are negatively correlated with resilience [49]. Findings that higher levels of resilience are protective regardless of the other three bullying roles [107] confirm the high levels of resilience we found in the low-risk group. Resilience is a vital personal resource that allows people to cope with or continue their lives despite negative impacts on their lives, such as cyberbullying [108]. Resilient adolescents can overcome cyberbullying experiences with coping resources [109]. Therefore, it may be helpful to focus on the development of protective personal factors such as resilience in protective and preventive studies related to cyberbullying in adolescents. Like resilience, past research has also shown that self-control protects against cyberbullying [53, 54]. It demonstrates that self-regulation is crucial for adolescents to combat cyberbullying [52]. Self-regulation has been identified as a protective factor buffering cyberbullying [55], and increasing self-efficacy is stated to be an essential factor in preventing bullying [110]. Examining social and emotional competencies is a promising approach that may shed new light on variables associated with participation in cyberbullying roles [111]. The findings obtained in this study showed that self-regulation, a vital competence, is protective for the low-risk cyberbullying profile. Based on the findings of this study, the low-risk group exhibited higher levels of resilience and self-regulation, which played a significant role in protecting against cyberbullying. This result aligns with previous research indicating that both resilience and self-regulation are crucial factors in preventing or mitigating cyberbullying experiences in adolescents. Therefore, developing these protective personal resources should be prioritized in future prevention and intervention programs to reduce cyberbullying.

Regarding self-regulation and resilience, the group referred to as the "medium risk group" in the study had the lowest scores compared to other groups. Another profile regarding cyberbullying in this study was the "high-risk group,” and they scored moderately compared to other groups regarding self-regulation and resilience scores. Findings showed that adolescents in the moderate-risk group demonstrated low self-regulation and resilience. In contrast, those in the high-risk group demonstrated moderate resilience and self-regulation. Studies have shown that increased stressors negatively impact processes that help with coping, such as self-regulation [112] and suggest that self-regulation promotes more problem-focused approaches to coping with stressful situations, increasing more resilient behaviors [113]. Resilience refers to adapting to stressful events [43] and is a protective factor against negative experiences such as cyberbullying, especially during adolescence [47, 48]. Students who exhibit better self-regulation demonstrated through their behavior, are better equipped to handle the stress associated with cyberbullying [114]. According to the findings of our study, while low levels of resilience and self-regulation were observed in individuals in the moderate-risk group, moderate levels of resilience and self-regulation in adolescents in the high-risk group can be attributed to the stress level caused by cyberbullying. The development of protective personal factors such as self-regulation and resilience requires individuals to experience a certain level of stress, leading to coping efforts [115]. Individuals facing stressful experiences can develop personal and social resources, such as resilience and self-regulation, by addressing problems [116–119] Individuals in the low-risk group may be less likely to participate in cyberbullying roles due to their high levels of resilience and self-regulation, which act as protective factors. On the other hand, individuals in the high-risk group may have developed resilience and self-regulation as a result of coping with the stress created by their bullying experiences. The findings indicate that adolescents in the moderate-risk group exhibited lower levels of resilience and self-regulation compared to their peers, which may stem from insufficient stress to activate coping mechanisms. While individuals in the high-risk group may develop these protective factors through direct experiences with stress, those in the moderate-risk group may not have encountered enough stress to foster the development of self-regulation and resilience. Future research should explore the threshold of stress necessary to trigger these protective responses and examine how moderate levels of exposure influence the development of coping skills in adolescence.

Consistent with the research findings on loneliness, resilience, and self-regulation, cyberbullying and cyberbullying profiles are theoretically interconnected with these constructs. In this context, the Social-Emotional Learning (SEL) framework provides a critical perspective, as it encompasses key concepts such as self-control, self-monitoring, and self-awareness, all of which serve as protective factors against potential risks in individuals’ lives [58]. Similarly, resilience, situated within the framework of Positive Youth Development (PYD), not only fosters the development of positive behaviors but also acts as a buffer against engagement in risky behaviors [47]. Furthermore, within the Socio-Ecological Theory [59], cyberbullying victimization is conceptualized as a consequence of complex social interactions, wherein factors such as loneliness and social connectedness play a pivotal role in shaping an individual's vulnerability to or resilience against such experiences. These variables, which are discussed in our research and are related to cyberbullying profiles, emphasize the multidimensional nature of the dynamics of cyberbullying in different ways and express the necessity of a multidimensional approach to understanding and reducing the risks associated with cyberbullying.

Lastly, in this study, we aimed to analyze the differences in cyberbullying profiles of being a cyberbully, cyber victim, cyber bystander, loneliness, self-regulation, and resilience in a sample of adolescents. To this end, we determined whether there were statistically significant differences between the groups regarding being a cyberbully, cyber victim, and cyber bystander, as well as loneliness, resilience, and self-regulation. The findings revealed that the scores related to the variables differed statistically among the determined profiles. Our findings showed that cyberbullying roles emerged in a similar parallel manner across the three profiles. This result confirms the literature in that all bullying roles manifest themselves in all three profiles, considering that being in any of the cyberbullying roles increases the likelihood of being in other roles [16, 80] and provides a more in-depth understanding of the literature. In this sense, it can be said that being a cyber bystander or a cyber victim is a risk factor, just like bullying cyberbullying behavior. Although the scores between the profiles regarding loneliness are close, they have been determined to differ statistically significantly. In particular, adolescents in the moderate-risk group had the highest loneliness scores. In contrast, adolescents in the high-risk group had lower scores than other groups, and finally, adolescents in the low-risk group had the lowest loneliness scores. These results are consistent with findings indicating that loneliness is a risk factor for cyberbullying [120, 121]. However, the lack of parallelism between loneliness levels according to risk level indicates that the role of loneliness in cyberbullying profiles should be examined in more depth. Additionally, the findings of this study may support previous claims that cyberbullying profiles are associated with a pattern of self-regulation and resilience [47, 48, 117]. However, it is believed that further explanation is needed for the complex relationship between perceived stress levels, individual factors related to perceiving bullying, and levels of resilience and self-regulation.

Bullying prevention programs integrated into school curricula are in a critical position to protect the mental health and academic life of individuals, especially those in adolescence, by including comprehensive training on cyberbullying [3]. Given the importance of teaching adolescents how to mitigate risks in cyberspace through the use of healthy coping strategies and seeking support from friends and family [122], It may be beneficial to incorporate the activation of these support mechanisms into school curricula. In this study, the data obtained regarding these variables offer an in-depth examination of three different cyberbullying profiles and provide an opportunity to understand better the relationship between cyberbullying and personal and social factors. Finally, considering that focusing more on roles such as cyber victim and cyber bystander, as well as adolescent individuals in the role of a cyberbully, can contribute to the prevention of bullying to a significant extent [29], it can be recommended that preventive intervention services focus on different profiles.

## Limitations and future directions

This study contributes to personality and clinical research by exploring the relationship between cyberbullying roles in adolescence and personal characteristics. However, it has limitations. While the sample size is adequate, future studies should use larger, more diverse samples. Since adolescence spans an extended period, the roles related to cyberbullying may differ, and its effects are long-term during this period. The cross-sectional design limits the findings to one point, so future research should take longitudinal measurements to observe long-term effects. Finally, this study focused on personal-social factors such as loneliness, self-regulation, and resilience when addressing cyberbullying profiles. The effects of cyberbullying on the mental health of children and adolescents need to be examined to develop counseling programs for cyberbullying victims [24]. Future research could focus on contextual, family factors and developmental processes that can potentially influence bullying profiles. The connections between these phenomena and individual personality differences should be explored in future research. In this research, cyberbullying profiles were examined for individuals in adolescence. Future studies can be conducted to examine cyberbullying profiles for individuals in different life stages. The variables in this study were characterized as individual profiles rather than just correlational relationships. Investigating the underlying reasons for the observations related to these profiles may be beneficial. For instance, despite the differing averages across all profiles, loneliness consistently displayed similar average scores in all three profiles. Additionally, as the study was conducted on a sample of normative Turkish adolescents, further research in Türkiye must clarify and expand upon the findings. Lastly, future research should focus on developing preventive and developmental psychological counseling practices that incorporate protective factors identified in low, medium, and high-risk groups in terms of cyberbullying profiles. Studies should be conducted to uncover the impact of resilience and self-regulation in reducing and preventing victimization among individuals in the high-risk group, and theoretical knowledge should be empirically tested.

## Conclusions

The study provides valuable insight for researchers looking to develop individual-centered research methods and address the influence of variables such as loneliness, self-control, and resilience in cyberbullying. It aims to expand current knowledge on cyberbullying roles and their correlation with personal and social factors. The results categorized adolescents into low-risk, moderate-risk, and high-risk groups for cyberbullying roles and revealed the protective influence of self-regulation and resilience. The findings indicate that resilience and self-regulation may protect adolescents from participating in cyberbullying roles, with the high-risk group showing moderate levels compared to the other two groups. Resilience and self-regulation are essential and should be considered in all cyberbullying roles for low and high-risk groups. The study's findings may advance researchers’ understanding of cyberbullying and are crucial for mental health professionals working in schools.

## Data Availability

No datasets were generated or analysed during the current study.

## Abbreviations

LPA: Latent profile analysis
LCA: Latent class analysis
